# Implementation of an Enhanced Recovery Pathway for Minimally Invasive Pectus Surgery: A Population-Based Cohort Study Evaluating Short- and Long-Term Outcomes Using eHealth Technology

**DOI:** 10.2196/10996

**Published:** 2018-10-12

**Authors:** Davina Wildemeersch, Michiel D'Hondt, Lisa Bernaerts, Pieter Mertens, Vera Saldien, Jeroen MH Hendriks, Anne-Sophie Walcarius, Lutgard Sterkens, Guy H Hans

**Affiliations:** 1 Department of Anesthesiology Antwerp University Hospital Edegem Belgium; 2 Multidisciplinary Pain Center Antwerp University Hospital Edegem Belgium; 3 Laboratory for Pain Research University of Antwerp Wilrijk Belgium; 4 Division of Psychology Multidisciplinary Pain Center Antwerp University Hospital Edegem Belgium; 5 Department of Thoracic and Vascular Surgery Antwerp University Hospital Edegem Belgium; 6 Department of Physical Medicine and Rehabilitation Antwerp University Hospital Edegem Belgium

**Keywords:** enhanced recovery, pectus carinatum, funnel chest, telemedicine, persistent postsurgical pain, mobile phone, eHealth, pediatric surgery, thorarcic surgery

## Abstract

**Background:**

Pectus excavatum and pectus carinatum are the most common chest wall deformities. Although minimally invasive correction (minimally invasive repair of pectus, MIRP) has become common practice, it remains associated with severe postoperative pain. Preoperative psychosocial factors such as anxiety and low self-esteem can increase postsurgical pain. Early detection of psychological symptoms, effective biopsychosocial perioperative management of patients, and prevention of pain chronification using an enhanced recovery pathway (ERP) may improve outcomes. However, the incidence of the latter is poorly described in adolescents undergoing MIRP.

**Objective:**

The objective of our study was to evaluate the implementation of an ERP containing early recovery goals and to assess persistent postsurgical pain 3 months postoperatively in pediatric patients undergoing MIRP. The ERP consists of a Web-based platform containing psychological screening questionnaires and extensive telemonitoring for follow-up of patients at home.

**Methods:**

A population-based cohort study was conducted with prospectively collected data from patients undergoing pectus surgery between June 2017 and December 2017. An ERP was initiated preoperatively; it included patient education, electronic health-based psychological screening, multimodal pre-emptive analgesia, nausea prophylaxis as well as early Foley catheter removal and respiratory exercises. After hospital discharge, patients were followed up to 10 weeks using a Web-based diary evaluating pain and sleep quality, while their rehabilitation progress was monitored via Bluetooth-connected telemonitoring devices.

**Results:**

We enrolled 29 adolescents using the developed ERP. Pre-emptive multimodal analgesia pain rating scores were low at hospital admission. Optimal epidural placement, defined by T8-9 or T9-10, occurred in 90% (26/29) of the participants; thus, no motor block or Horner syndrome occurred. Mean bladder catheterization duration was 3.41 (SD 1.50) days in ERP patients. Numeric rating scale (NRS) scores for pain and the incidence of nausea were low, contributing to a fluent rehabilitation. Mean NRS scores were 2.58 (SD 1.77) on postoperative day (POD) 1, 2.48 (SD 1.66) on POD 2, and 3.14 (SD 1.98) on POD 3 in ERP-treated patients. Telemonitoring at home was feasible in adolescents after hospital discharge despite adherence difficulties. Although the pain scores at the final interview were low (0.81 [SD 1.33]), 33% (9/27) long-term follow-up ERP patients still experienced frequent disturbing thoracic pain, requiring analgesic administration, school absenteeism, and multiple doctor (re)visits.

**Conclusions:**

Allocating patients to the appropriate level of care preoperatively and immediately postoperatively may improve long-term outcome variables. Internet-based technologies and feasible, objective monitoring tools can help clinicians screen surgical patients for risk factors and initiate early treatment when indicated. Future research should focus on improving risk stratification and include a psychological assessment and evaluation of the effect of perioperative care pathways in children undergoing major surgery.

**Trial Registration:**

ClinicalTrials.gov NCT03100669; https://clinicaltrials.gov/ct2/show/NCT03100669 (Archived by WebCite at http://www.webcitation.org/72qLB1ADX)

## Introduction

Funnel chest (pectus excavatum, PE) occurs in 1 out of 400-1000 live births and is the most common chest wall deformity (80%-90% incidence rate); additionally, it affects 4 times more males than females. Pectus carinatum (PC) is the second most common anterior chest deformity (15%), with an even more pronounced male predominance [1]. Surgery, frequently during childhood, is often planned for esthetic reasons rather than as a necessary correction due to compression of underlying organs. Although minimally invasive correction (minimally invasive repair of pectus, MIRP) has become common practice because of the reduced surgical stress response, lower blood loss, and smaller incisions [2], it remains associated with severe acute and persistent postoperative pain. Psychosocial factors, including preoperative anxiety and low self-esteem, are identified as risk factors for increased postoperative pain [3-5]. Furthermore, evidence has revealed that patients undergoing thorax surgery are prone to the development of persistent postsurgical pain (PPSP) [6,7], which is often neuropathic and, therefore, more difficult to treat. However, little is currently known about the precise incidence of PPSP in children after pectus surgery. Despite the increased scientific interest in pain management after pectus surgery [8,9], the provision of adequate pain management and the necessary antiemetic and psychological treatments during the whole perioperative period remain a challenge for health care providers.

Recently, enhanced recovery pathways (ERPs) have been implemented worldwide as evidence-based standardized perioperative approaches. ERPs became the standard of care for patients undergoing colorectal surgery [10]. By introducing enhanced recovery programs, multidisciplinary teams began working together, and the traditional care model was shifted to a more holistic approach, improving many patient-related outcome measurements by reducing the variation of care. The implementation of such ERPs for children and adolescents undergoing MIRP may not only reduce postoperative acute pain and increase overall satisfaction but also provide early alerts to caregivers regarding potential risk factors for increased postoperative pain or PPSP, allowing early treatment that may further improve patient outcomes. The use of one of the most rapidly growing health care innovations [11], electronic health (eHealth) technology (smartphone apps, individual Web-based platforms, and medical devices), may facilitate biopsychosocial follow-up, especially in the long term after hospital discharge [12].

In this study, we aimed to evaluate the implementation of a newly developed holistic ERP for adolescents undergoing elective MIRP surgery utilizing eHealth technology for preoperative psychological screening and long-term postoperative patient follow-up.

## Methods

### Recruitment Enhanced Recovery Pathway-Treated Patients

Between June 2017 and December 2017, 29 patients scheduled for MIRP were managed via the implemented multidisciplinary perioperative care pathway after obtaining written informed consent. All surgical procedures were performed by one attending pediatric thoracic surgeon. The technique used has been described by Nuss et al for PE [2] and by Abramson et al for PC [13]. Patients with a history of psychiatric disease, chronic opioid use (>3 months), or revision surgery were excluded from this implementation study. All patients were recruited by the Department of Thoracic and Vascular Surgery and, subsequently, selected for this study by the Anesthesiology Department, Antwerp University Hospital, Belgium. Notably, 2 patients refused preoperative psychological screening via Web-based questionnaires and long-term follow-up via individual eHealth technology. None of the patients reported preoperative pain symptoms. Questionnaire reports and medical data obtained before and after hospital admission were recorded by patients via a specifically designed electronic medical record, supporting an individualized approach.

This population-based cohort study was performed in accordance with the ethical standards of International Conference on Harmonisation-Good Clinical Practice and the Declaration of Helsinki after obtaining study approval from the Institutional Review Board (IRB) and Ethics Committee of the Antwerp University Hospital, Belgium (study identifier: 17/08/082) and trial registration (ClinicalTrials.gov NCT03100669). No additional specific IRB approval was requested for the retrospective control cohort as such retrospective use of patient data is already fully covered by a waiver granted by the general IRB that is applicable within the hospital for all research-related activities. The existence of this general IRB was made known to each patient upon admission to hospital, and approval was obtained from each patient. The specifics of the data extraction performed within this retrospective cohort were submitted to the EC for acknowledgment and filing.

### Historical Controls

This paper reports initial findings after the implementation of an ERP in patients undergoing pectus surgery in the Antwerp University Hospital, Belgium. Results of this implementation study were analyzed and compared with retrospective acquired administrative data collected from medical charts and hospital records. The retrospectively derived control patient cohort at our hospital underwent identical pectus procedure by the same surgeon without an ERP and were selected by age (≤18 years) and pathology (PE and PC).

### Multidisciplinary Enhanced Recovery Pathway

**Figure 1:**
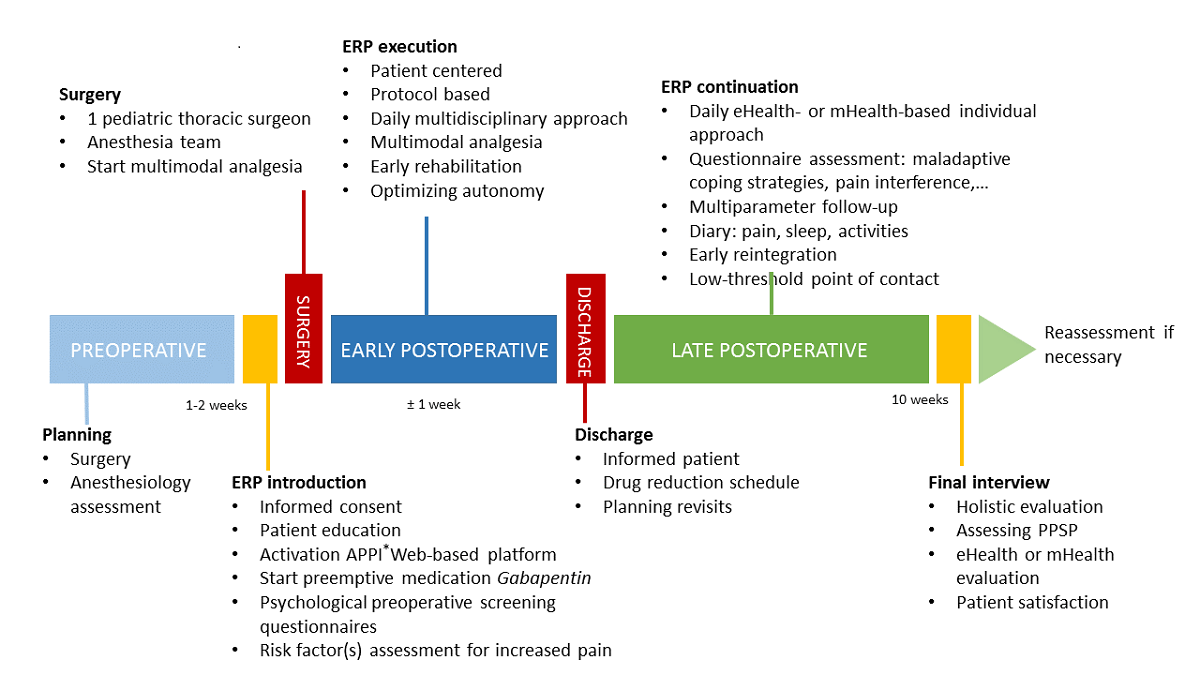
Protocol design—timeline. ERP: enhanced recovery pathway; APPI: Antwerp Personalized Pain Initiative; PPSP: persistent postsurgical pain.

**Figure 2:**
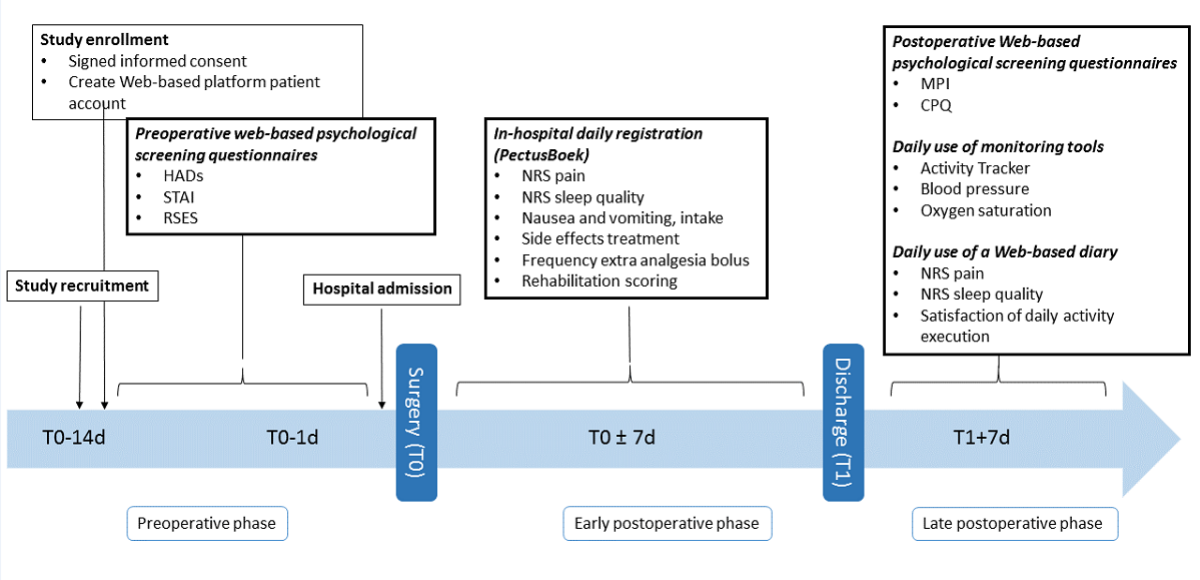
Timeline of the conducted surveys. T0: day of surgery; T1: day of hospital discharge; HADs: Hospital Anxiety and Depression Scale; STAI: State-Trait Anxiety Inventory; RSES: Rosenberg Self-Esteem Scale; NRS: Numeric Rating Scale; MPI: Multidisciplinary Pain Inventory; CPQ: Coping Pain Questionnaire.

Figures 1 and 2 present the components of the multidisciplinary ERP.

### Preoperative Study Phase

**Figure 3:**
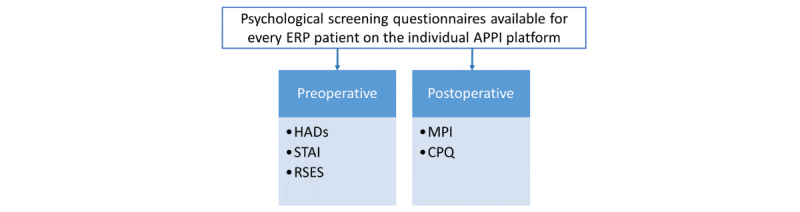
Multidisciplinary enhanced recovery pathway—psychological elements. ERP: enhanced recovery pathway; APPI: Antwerp Personalized Pain Initiative; HADs: Hospital Anxiety and Depression Scale; STAI: State-Trait Anxiety Inventory; RSES: Rosenberg Self-Esteem Scale; MPI: Multidimensional Pain Inventory; CPQ: Coping with Pain Questionnaire.

A clinical study interview was executed 1-2 weeks preoperatively. A preoperative psychological inventory [14] was performed by patients after activation of the personal Web-based Antwerp Personalized Pain Initiative (APPI; Appi@Home, a European Union registered trademark under registration #017610627) platform; Figure 3; Multimedia Appendix 1). Validated Web-based Dutch questionnaires (Multimedia Appendix 2) included screening for anxiety and depressive symptoms (*Hospital Anxiety and Depression Scale,* HADs [15]), or trait characteristics (*State-Trait Anxiety Inventory,* STAI [16]) and self-esteem (*Rosenberg Self-Esteem Scale*, RSES [17]). Self-assessment through the abovementioned Web-based questionnaires were used in this Web-based trial part. If deviating or alarming questionnaire scores were recorded, an appointment with the psychologist was scheduled preoperatively. In addition, alarming scores were defined on normative data and described cutoffs, as previously described [14]. If present, the appropriate treatment was performed by a specialized psychologist.

The routine preanesthetic assessment included taking patient history and performing clinical examination, blood collection, and technical cardiac and pulmonary investigations if necessary, supplemented by an extensive information session regarding the anticipated surgical trajectory. Key features regarding postoperative pain, pain management with patient-controlled thoracic epidural analgesia (PCEA), and the Foley catheter were included in a procedure-specific information leaflet. Preoperative assessment included the administration of a 7-day regimen of oral gabapentin 1 week preoperatively and alignment of patients’ expectations.

### Early Postoperative Study Phase

Multimedia Appendix 3 provides a complete overview of the used ERP protocol during hospital admission. In brief, the intraoperative treatment included multimodal analgesia using a thoracic epidural opioid-local anesthetic mixture, ketorolac, and acetaminophen based on patient weight. Additionally, the ERP featured a maximal multimodal antiemetic strategy including dexamethasone, ranitidine, dehydrobenzperidol, and propofol for anesthesia maintenance. Immediately after surgery, patients were admitted to the postanesthesia care unit and were transferred to the ward when postanesthesia care unit discharge criteria were fulfilled. Postoperatively, oral gabapentin was continued for ERP patients in addition to PCEA, nonsteroidal anti-inflammatory drugs, and acetaminophen around-the-clock. The use of intravenous morphine or tramadol was strictly avoided, and a rigorous antiemetic strategy included ondansetron administration during the PCEA regimen. If necessary, escape analgesia for breakthrough pain and antiemetic rescue was available. In the subsequent days, PCEA settings were decreased in a stepwise fashion according to the protocol. Implementation of a programmed intermittent bolus regimen was applied to diminish rebound pain during the reduction of the PCEA dose. Under the protocol, PCEA was discontinued on postoperative day (POD) 6, or, if possible, on POD 5. Urinary catheters were removed as quickly as possible. During hospital admission, daily pain scores, respiratory rehabilitation, and vomiting were recorded in a multidisciplinary fashion. Nausea was noted when persistent. Patients were discharged on acetaminophen, a fixed combination of tilidine and naloxone (Valtran Retard), and gabapentin. Upon discharge from the hospital, patients were provided with a reduction scheme for the analgesic intake over a period of 2 weeks (Multimedia Appendix 4).

### Late Postoperative Study Phase

The extended ERP included a follow-up period of 10 weeks postoperatively to meet the PPSP working definition proposed by Werner and Kongsgaard [18]. After hospital discharge, 2 Web-based questionnaires were provided for completion within the first week after hospital admission to screen for maladaptive coping strategies and pain-rehabilitation interference using their individual Appi@Home platform.

**Figure 4:**
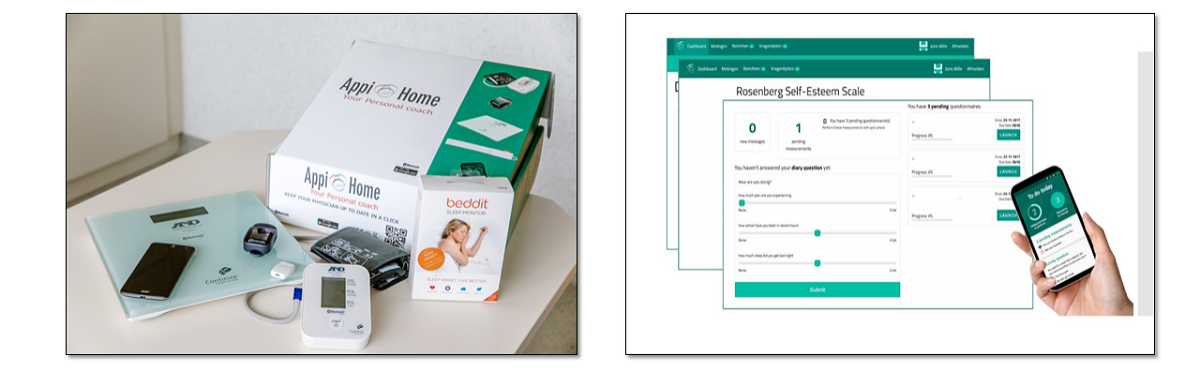
The Appi@Home toolbox and smartphone app—the medical devices for patient monitoring after hospital discharge.

Scores of the validated Dutch questionnaires (Multimedia Appendix 5) from the Multidimensional Pain Inventory (MPI) [19] and the Coping Pain Questionnaire (CPQ) [20] were assessed. Using eHealth technology, adolescents used their smartphones to log in to the Appi@Home smartphone app for the direct transmission of the derived objective parameters of 3 medical-rated telemonitoring devices (activity tracker, blood pressure monitor, and oxygen saturation measurement device) in the ubiquitous health monitoring system Appi@Home (Figure 4; Multimedia Appendix 1). In addition, the objective data were supplemented by subjective personal diary answers, including daily pain, sleep, and activity assessments on an 11-level scale, which was asked to be filled in daily via the Appi@Home app on patients’ smartphones. When no (objective or subjective) data were obtained for 1 week, patients received a single reminder via the platform. If no response was provided, patients were contacted via telephone and asked about their well-being; furthermore, measurement instructions were repeated and patients were noted as nonadherent. Adherence is referred to as the capacity of a patient to abide by mutually agreed recommendations regarding daily monitoring [20,21]. Patients presented for postoperative evaluation visits 1-2 weeks after surgery and 2-3 months after surgery at the Department of Thoracic Surgery according to surgeon preference.

The final study interview was planned 3 months postoperatively for patients on an ERP. In-hospital reassessments were scheduled earlier if necessary. An integrated final assessment was executed by a study physician or team member from the multidisciplinary pain center. Furthermore, the intake of medication and side effects, the presence of sleep disturbances, presence of PPSP, school absenteeism, and overall satisfaction were recorded. Moreover, a thorough evaluation of the Web-based platform was performed.

### Data Analysis

All data were recorded using a specific designed, multidisciplinary registration tool (“PectusBoek”) and Microsoft Excel for Windows 2016 (Microsoft Corporation, Redmond, WA). Patient characteristics were extracted from the electronic patient record (C-medical record, Cegeka, Vienna, Austria) during the hospital stay. In addition, questionnaire scores, diary answers, and medical devices data were derived from their individual eHealth APPI platforms and described. Data were analyzed using SPSS Statistics software, version 21.0 for Windows (IBM Corp, Armonk, NY, United States).

Numeric Rating Scale scores (NRS) for pain and nausea symptoms and subjective sleep scores were summarized and described. When multiple pain scores were assessed in a single day, the day’s scores were averaged. A supplementary NRS was recorded by a specialized pain nurse, as were PCEA-related side effects or complications. Furthermore, rehabilitation measures, including flow-oriented spirometry and posture exercises, were evaluated and recorded by a specialized physiotherapist.

Values for the postoperative length of hospital stay (LOS), days of PCEA, and urinary catheterization of patients on an ERP were compared with the corresponding values in the cohort of the previous 93 (ratio 1:3 to reduce selection bias) adolescent pectus procedure patients at our institution before the ERP transition period. The relationships between patient characteristics and outcome variables were analyzed using the independent sample *t* test and chi-square test after normality control.

## Results

### Patient Characteristics

Overall, 28 males and 1 female (age range 12-18 years) underwent MIRP via the ERP protocol. Of them, 23 were treated for a PE deformity. The mean Haller Index was 3.53 (range 2.5-6.8); however, this outcome was measured in only 9 of 23 patients with PE. Mean body length and body mass index were 174.28 (SD 9.14) cm and 18.37 (SD 2.30) kg/m^2^, respectively.

### Early Recovery: Pain Assessment and Related Outcome Variables

Nausea symptoms were reduced in ERP patients on POD 1 compared with previously operated patients undergoing the same procedure at our hospital, as indicated by their data (5/29, 17%, ERP participants vs 37/93, 40%, non-ERP-treated patients; *P*=.03). Of the 29 ERP-treated patients, 1 (3%) reported nausea symptoms more than once the day after surgery. The highest incidence of postoperative nausea among patients using the ERP was recorded on POD 3 in 24% (7/29) participants, and 10% (3/29) of them reported nausea symptoms more than twice that day, despite multimodal antiemetic strategies. In 2 ERP patients, nausea was associated with vomiting.

If other side effects were present during the ERP treatment, pruritus was most frequent (25/29, 86%) during the PCEA administration, followed by dizziness (4/29, 14%) within the first 3 PODs. Not unexpectedly, ERP patients had a significantly less neuraxial analgesia side effect (1/29, 0.3%, ERP patients vs 20/93, 22%, non-ERP patients; *P*=.03) after the standardized thoracic catheter insertion; furthermore, accurate pain reduction was reflected in a longer PCEA administration period for ERP patients (5.76 [SD 1.02] days vs 4.67 [SD 1.20] days; *P*<.001]. Enrolled ERP patients followed the PCEA weaning protocol, and PCEA was discontinued in 38% (11/29) patients on POD 5 and in 90% (26/29) patients on POD 6. PCEA characteristics were compared with previous non-ERP-treated patients at our hospital (Table 1). Using the 11-level NRS pain scale (0: no pain to 10: worst pain), average pain scores given by the educated patients are shown in Table 2.

Of all ERP participants, 64% (18/29) were able to maximally execute flow-oriented incentive spirometry on POD 1, 93% (25/29) on POD 2, and all of them on POD 3. In addition, 30% (8/29) patients were able to execute physical exercises while standing upright on POD 2; this number increased during the consecutive days to 67% (18/29) on POD 3, 77% (20/29) on POD 4, and 96% (26/29) on POD 5. Moreover, patients were stimulated to increase mobilization and walk from POD 3 onward. Furthermore, 26% (7/29) patients were able to walk on POD3, 58% (15/29) on POD 4, and 82% (22/29) on POD 5. However, no rehabilitation data were available for patients treated without a standardized perioperative protocol.

**Table 1:** Patient-controlled epidural analgesia (PCEA) characteristics in patients undergoing minimally invasive repair of pectus with and without an enhanced recovery pathway (ERP).

Postoperative day		ERP-treated patients (n=29)	Non-ERP-treated patients (controls; n=93)	*P* value
**Thoracic-level PCEA, n (%)**				<.001
	T8-10	26 (90)	0 (0)	
	Other	3 (10)	93 (100)	
**Problem^a^, n (%)**				.03
	Yes: no	1:28 (0.3)	20:73 (22)	
	Horner syndrome	0 (0)	12 (60)	
	Motor blockade	0 (0)	3 (15)	
	Prematurely removed	1 (0.3)	5 (25)	
Length of PCEA, mean (SD)		5.76 (1.02)	4.67 (1.20)	<.001

^a^Problem defined as Horner syndrome, motor blockade, or unforeseen premature PCEA discontinuation.

**Table 2:** Average pain scores assessed by a specialized pain care provider in patients treated with and without an enhanced recovery pathway (ERP).

Postoperative day (POD)		ERP-treated patients (n=29), mean (SD)	Non-ERP-treated patients (controls; n=93), mean (SD)	*P* value
**POD 1**				
	At rest	1.26 (1.43)	1.24 (1.40)	.94
	During exercise	2.58 (1.77)	2.84 (1.60)	.50
**POD 2**				
	At rest	1.08 (1.38)	1.41 (1.62)	.36
	During exercise	2.48 (1.66)	3.24 (1.70)	.05^a^
**POD 3**				
	At rest	1.58 (2.15)	1.16 (1.16)	.37
	During exercise	3.14 (1.98)	2.66 (1.40)	.19
**POD 4**				
	At rest	1.73 (1.76)	1.29 (1.74)	.26
	During exercise	3.71 (2.16)	2.70 (1.79)	.02^a^
**POD 5**				
	At rest	1.52 (1.87)	1.00 (1.59)	.16
	During exercise	2.84 (1.70)	2.23 (1.69)	.12

^a^Significant at *P<*.05.

**Figure 5:**
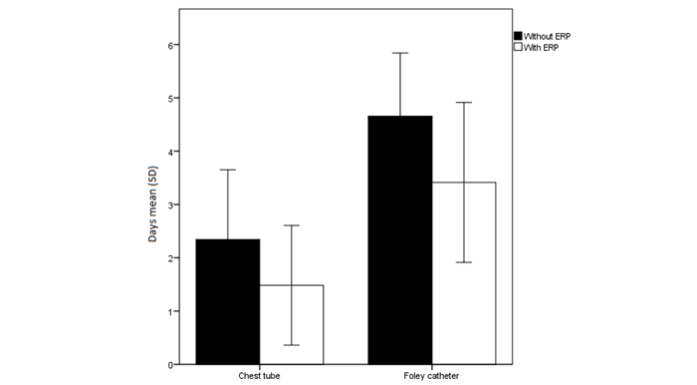
The chest tube and urinary catheter duration (mean [SD]) in patients treated with and without an enhanced recovery pathway (ERP).

ERP-treated patients had a significantly reduced Foley catheterization period (3.41 [SD 1.50] vs 4.66 [SD 1.18] days; *P*<.001) with a much sooner removal of the chest tube (1.48 [SD 1.12] vs 2.34 [SD 1.31] days; *P*=.002; Figure 5) compared with non-ERP-treated patients, as indicated by their retrospective data, at our hospital. However, the LOS was longer in the ERP-treated group (7.66 [SD 2.01] vs 6.32 [SD 1.26] days; *P*<.001]. ERP-treated patients could have been discharged after 6.59 (SD 1.99) days (*P*=.40), but they stayed in the hospital for diverse nonmedical reasons.

### Early Psychological Screening in Surgical Patients Treated With the Enhanced Recovery Pathway

The implementation of psychological screening tools is an innovative feature of the ERP protocol. The PPSP-defined risk factors for anxiety, depression, and low self-esteem were identified using 3 Web-based questionnaires before surgery. Table Questionnaire scores and normative “control” data are summarized in Table 3.

The HADS has been developed for detecting states of depression and anxiety in a hospital setting [22,23]; it contains 2 subscales to assess the presence of an anxiety or depressive disorder. The overall mean score for “fear” was 6.00 (SD 3.20; range: 1-12), indicating the absence of anxiety states prior to surgery. In addition, 71% (17/29) patients scored between 0 and 7 (no anxiety), and 21% (5/29) patients scored between 8 and 10 (possible anxiety); 8% (2/29) patients scored ≥11 (probable anxiety). Screening for depressive disorders showed a mean score of 3.33 (SD 2.76; range: 0-10) and indicated the absence of depressive states prior to surgery. Moreover, 92% (22/29) patients scored 0-7 (no depression), and 8% (2/29) patients scored 8-10 (possible depression). No patient with an alarming score was identified by either subscale. Additionally, trait anxiety was measured using the STAI-DY-2. The overall mean score of the study sample (38.67 [SD 7.99]) was compared with available control data of a group of 18-year-old male military recruits (decile 6) [24], which indicated a mean level of trait anxiety in the enrolled ERP patients.

For evaluation of global self-esteem in patients undergoing MIRP with an ERP, the RSES was used. The RSES is a screening instrument for negative body image perception [25]. The mean score of the overall patient sample was 21.25 (SD 3.49), which was above the theoretically defined cutoff score of 15 [26]. No single patient scored beneath this cutoff. On comparing mean self-esteem levels across 53 nations, we found higher self-esteem among our patients than among Belgian patients with a mean score of 19.66 (SD 5.28) [26].

The MPI measures various pain-relevant aspects. We focused on the “pain severity” and “interference” subclasses; therefore, the Dutch version of the MPI questionnaire was used [19]. The mean score of the study sample was compared with the available normative data (mean and SD) of the “IASP Primary Site: Thoracic Region” [27]. The overall mean “pain severity” score in our patients was 2.27 (SD 1.09), which was lower than that of the normative sample (5.01 [SD 0.82]). The overall mean “pain interference” score in our patients was 3.41 (SD 0.81), which was also lower than that of the normative sample (5.01 [SD 0.80]).

For assessing various pain-coping strategies, the CPQ was used [28]. CPQ active and passive coping indices were calculated according to the method described by Soares and Grossi [29] and Nicholas et al [30]. The mean raw subscale scores were compared with those of the normal group of patients with chronic low back pain or neck pain because an identical control group was missing [31]. The decile scores are written in parentheses below. The overall mean “diverting attention” score was 21.32 (SD 12.89; decile 4). The overall mean “reinterpret pain sensation” score was 8.18 (SD 6.41; decile 2). The overall mean “catastrophizing” score was 10.45 (SD 8.96; decile 2).

**Table 3:** Detailed questionnaire scores from Web-based psychological screening.

Questionnaire variables			Questionnaire outcome	Available data^a^
**Hospital Anxiety and Depression Scale**				
	**Fear, mean (SD)**		6.00 (3.20)	—^b^
		No anxiety, n (%)	17 (71)	Cutoff: ≤7
		Possible anxiety, n (%)	5 (21)	Cutoff: ≥8, but <10
		Probable anxiety, n (%)	2 (8)	Cutoff: ≥10
	**Depression, mean (SD)**		3.33 (2.76)	—
		No depression, n (%)	22 (92)	Cutoff: ≤7
		Possible depression, n (%)	2 (8)	Cutoff: ≥8, but <10
		Probable depression, n (%)	0 (0)	Cutoff: ≥10
**State-Trait Anxiety Inventory, mean (SD)**				
	Trait anxiety		38.67 (7.99)	Decile 6
Rosenberg Self-Esteem Scale, mean (SD)			21.25 (3.49)	Midpoint cutoff: 15
**Multidimensional Pain Inventory, mean (SD)**				
	Pain severity		2.27 (1.09)	5.01 (0.82)
	Pain interference		3.41 (0.81)	5.01 (0.80)
**Coping Pain Questionnaire, mean (SD)**				
	Diverting attention		21.32 (12.89)	Decile 4
	Reinterpret pain sensation		8.18 (6.41)	Decile 2
	Catastrophizing		10.45 (8.96)	Decile 2
	Ignore pain sensation		23.09 (12.44)	Decile 3
	Praying or hoping		20.00 (15.37)	Decile 5
	Coping self-statements		38.09 (11.52)	Decile 5
	Increased behavioral activities		19.95 (10.26)	Decile 3
	Perceived pain control		10.65 (5.69)	Decile 7

^a^Normative data and cutoff scores from previous literature, see text for references.

^b^No data available.

The overall mean “ignore pain sensation” score was 23.09 (SD 12.44; decile 3). The overall mean “praying or hoping” score was 20.00 (SD 15.37; decile 5). The overall mean “coping self-statements” score was 38.09 (SD 11.52; decile 5). The overall mean “increased behavioral activities” score was 19.95 (SD 10.26; decile 3). The overall mean “perceived pain control” score was 10.65 (SD 5.69; decile 7). Note that these scores represent the pain-coping ability of the study sample. The mean postoperative pain during the first week after discharge was low (NRS: 3.68 [SD 0.22]; MPI pain severity: 2.27 [SD 1.09]), reflecting the need to develop strategies to cope with pain.

### Long-Term Rehabilitation: Subjective and Objective Variables

There was a large variability in the use of the telemonitoring devices in the study sample. As patients were asked to use the devices every day during the 10-week follow-up period, we would theoretically receive, at least, 70 results from each patient’s monitoring tool when the patients’ adherence was maximal. On average, patients used the devices half as much as expected—only 38 times (Table 4).

There was very little evidence of vital sign problems in the study group (Multimedia Appendix 6), even during the first week when opioids were prescribed. Mean oxygen saturation, heart rate, and systolic blood pressure were 97.85% (SD 1.06%; range: 93%-100%), 81.69 (SD 12.60) beats per minute (range: 55-112), and 111.72 (SD 9.99) mm Hg (range: 90-159), respectively, during the first week after discharge. No alarming vital signs, defined as a systolic blood pressure <95 mm Hg or >140 mm Hg, oxygen saturation <95%, tachycardia >140 beats per minute, bradycardia <45 beats per minute, or >10% deviation from the last parameter control before hospital discharge, were recorded during the long-term study follow-up. These findings further indicate the overall wellness of patients after their discharge from the hospital.

**Table 4:** Per patient use of coupled telemonitoring devices that were asked to be actively used once a day and use of an eDiary in the follow-up period.

Parameter	Times used per patient, range	Mean (SD)
Oxygen saturation monitor	8-77	38.00 (21.93)
Blood pressure monitor	7-78	38.50 (23.12)
Diary	1-67	19.88 (16.03)

**Table 5:** Mean Numeric Rating Scale (NRS) scores for pain, rehabilitation, and sleep quality of enhanced recovery pathway patients after hospital discharge.

Weeks at home^a^	Results, n^b^	Pain^c^, mean (SD)	Daily activity^d^, mean (SD)	Sleep quality^e^, mean (SD)
Week 1 (≤7 days)	97	3.68 (0.22)	4.54 (0.19)	6.10 (0.22)
Week 2 (day 8-14)	70	3.14 (2.34)	5.29 (2.57)	5.29 (2.54)
Week 3 (day 15-21)	58	2.62 (1.92)	4.43 (2.42)	5.93 (2.26)
Week 4 (day 22-28)	52	2.71 (2.39)	5.54 (2.36)	6.40 (2.33)
Week 5 (day 29-35)	50	1.92 (1.88)	5.52 (3.13)	6.80 (2.52)
Week 6 (day 36-42)	38	1.89 (1.57)	6.03 (2.92)	6.50 (2.85)
Week 7 (day 43-49)	35	1.91 (2.37)	5.51 (3.04)	5.77 (3.26)
Week 8 (day 50-56)	25	2.60 (2.55)	5.40 (2.83)	6.36 (2.77)
Week 9 (day 57-63)	25	2.24 (2.28)	5.24 (2.79)	6.16 (2.78)
Week 10 (day 64-70)	17	2.18 (1.38)	6.06 (2.14)	7.41 (2.60)

^a^Results were collected using the Web-based platform during the defined follow-up period of 10 weeks postoperatively.

^b^Number of recorded measurements.

^c^0: no pain; 10: worst pain.

^d^0: worst activity execution possible; 10: ideal activity execution.

^e^0: worst sleep quality; 10: optimal sleep quality.

Mean NRS scores for pain intensity, daily activity execution, and subjective sleep quality within the first week of hospital discharge were 3.68 (SD 0.22), 4.54 (SD 0.19), and 6.10 (0.22), respectively. Table 5 gives an overview of the overall mean pain scores, daily activity execution capabilities, and subjective sleep quality during out of the hospital follow-up. All of these parameters favorably evolved in each patient during the postoperative phase (Multimedia Appendix 7), with decreasing pain scores and increasing scores for sleep quality and satisfaction with the performance of daily activities.

**Figure 6:**
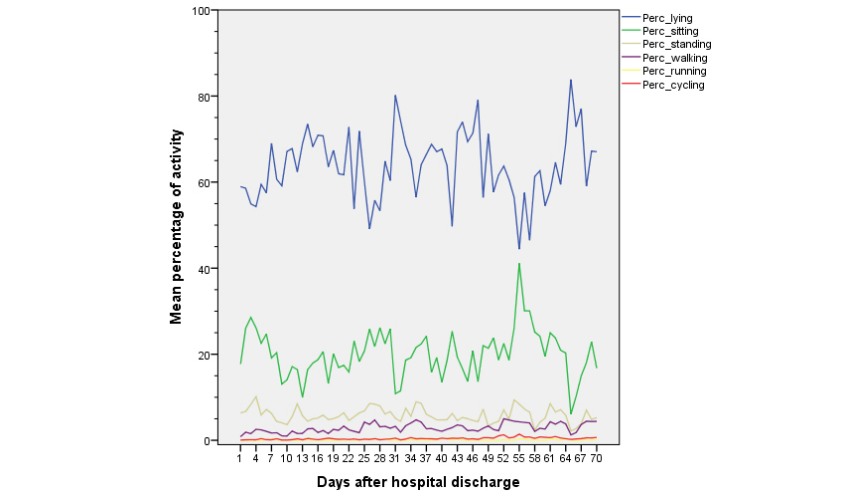
Study population mean objective activity variables during postoperative rehabilitation after hospital admission. Data are shown as mean percentages of daily activity evaluated in 6 categories: lying (blue), sitting (green), standing (dark yellow), walking (purple), running (yellow), and cycling (red).

**Figure 7:**
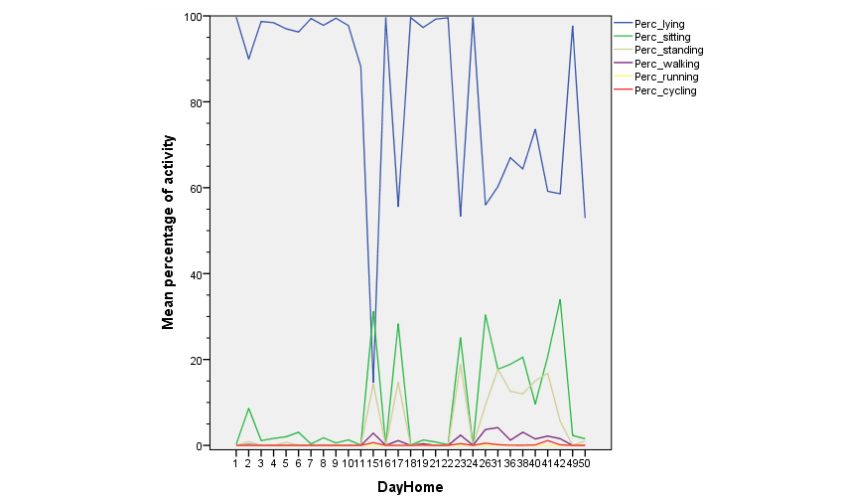
Evolution of daily activities during rehabilitation. Mean objective activity variables of patient Y.J. during postoperative rehabilitation after hospital admission. Data are given as mean percentages of daily activity evaluated in 6 categories; lying (blue), sitting (green), standing (dark yellow), walking (purple), running (yellow), and cycling (red).

Mean results from daily patient activity generated by the objective activity tracker are shown in Figure 6. The expected long-term postoperative rehabilitation is given in Figure 7, which is shown by the activity tracker data from patient YJ.

Overall, 24 patients used the activity tracker monitoring tool (Table 6). Results were registered in 6 different categories: lying, sitting, standing, walking, running, and cycling. The patients were able to track their activity during 39.79 (SD 5.12) days after surgery, with a large range in the patient individual monitoring use (minimum 1 day, up to maximal use during the study period). Theoretically, the 29 included ERP patients carried the activity tracker during, at least, 70 days, generating activity measurements during a total of 1890 days. During this ERP implementation study, the activity of ERP patients was tracked solely for 955 days (955/1890, 51%). Moreover, only 873 tracked days were evaluated as representative data; that is, activity day logs containing 24 hours of “lying” were interpreted as “tracker not used” and were excluded for data analysis. Patients were registered as “lying down” most frequently during the day. Moreover, “lying down” frequency did not decrease during the consecutive weeks after hospital discharge. Not surprisingly, patients seldom performed more intense activities such as running or cycling during the follow-up period.

No single patient-reported side effect from the perioperative intake of oral gabapentin was observed. In addition, 77% (20/26) patients did not report any side effects from the oral opioid administration on the final interview. When asked about symptoms, 4 patients reported drowsiness, and all others reported dizziness. All of these symptoms disappeared after dose reduction during the first 2 weeks after their hospital discharge.

Although mean pain scores were extremely low at the final interview (NRS: 0.81 [SD 1.33]), 11% (3/27) participants continued to use analgesics on a routine basis. Moreover, 37% (10/27) MIRP operated patients still experienced frequent disturbing pain 10 weeks postoperatively, leading to sporadic intake of analgesic drugs, school absenteeism, and multiple doctor (re)visits. All patients located the pain in the midaxillary thoracic region (5 patients even reported bilateral pain) and all described neuropathic pain characteristics.

**Table 6:** Mean activity levels in 6 different intensity categories registered by the activity monitoring tool over 24 hours per week after hospital discharge.

Weeks at home^a^	Days, n^b^	Lying		Sitting		Standing		Walking		Running		Cycling	
		Day, %	Hours, mean (SD)	Day, %	Hours, mean (SD)	Day, %	Hours, mean (SD)	Day, %	Hours, mean (SD)	Day, %	Hours, mean (SD)	Day, %	Hours, mean (SD)
Week 1	123	59.10	14.18 (6.30)	23.51	5.64 (4.21)	7.27	1.74 (1.65)	1.86	0.45 (0.46)	0.07	0.02 (0.12)	0.16	0.04 (0.06)
Week 2	121	65.44	15.71 (6.78)	15.29	3.67 (3.69)	5.08	1.22 (1.80)	1.65	0.40 (0.612)	0.05	0.01 (0.05)	0.21	0.05 (0.12)
Week 3	115	66.42	15.94 (5.91)	17.78	4.27 (3.86)	5.38	1.29 (1.31)	2.34	0.56 (0.72)	0.04	0.01 (0.03)	0.30	0.07 (0.14)
Week 4	80	60.01	14.40 (5.82)	21.47	5.15 (3.85)	6.76	1.62 (1.71)	3.07	0.74 (0.84)	0.12	0.03 (0.10)	0.23	0.05 (0.09)
Week 5	84	67.16	16.12 (5.57)	18.55	4.45 (3.90)	6.32	1.52 (1.50)	3.35	0.80 (0.82)	0.09	0.02 (0.09)	0.35	0.09 (0.17)
Week 6	79	64.25	15.42 (6.22)	19.51	4.68 (4.02)	5.79	1.39 (1.40)	2.81	0.67 (0.68)	0.16	0.04 (0.15)	0.39	0.09 (0.15)
Week 7	61	70.16	16.84 (7.01)	18.33	4.40 (4.56)	4.94	1.19 (1.53)	2.84	0.68 (0.77)	0.06	0.02 (0.72)	0.44	0.11 (0.17)
Week 8	51	57.68	13.84 (5.61)	25.35	6.08 (4.30)	6.25	1.50 (1.21)	3.76	0.90 (0.75)	0.12	0.03 (0.86)	0.86	0.21 (0.21)
Week 9	57	58.08	13.94 (6.92)	24.01	5.76 (5.45)	5.77	1.38 (1.49)	3.41	0.82 (0.83)	0.11	0.03 (0.07)	0.68	0.16 (0.21)
Week 10	46	70.37	16.89 (6.01)	15.69	3.76 (3.93)	4.62	1.11 (1.31)	3.41	0.82 (0.82)	0.12	0.03 (0.11)	0.44	0.11 (0.15)

^a^Results were collected using the Web-based platform during the defined follow-up period of 10 weeks postoperatively.

^b^Overall number of included measurement days.

Questions regarding Appi@Home satisfaction were asked at the final interview, 3 months postoperatively (Table 7) in this ERP implementation trial. In addition, 27 ERP-treated patients rated the smartphone app, the individual Web-based platform usability, and the platform accessibility as “good” or “excellent” in 78% (21/27), 85% (23/27), and 89% (24/27) cases, respectively. No individual scored the platform usability or accessibility as “insufficient.” Regarding the time burden for psychological assessments, 56% (15/27) participants indicated a (rather) low effort for questionnaire completion, and 19% (5/27) patients mentioned that an average effort was required. Overall, 78% (21/27) ERP patients were able to complete the Web-based questionnaires within the imposed deadlines.

The overall satisfaction after ERP was high. Of note, 17 patients rated the in-hospital care as “very good” and 8 rated it as “good,” and only 1 patient evaluated the overall care as “sufficient.” The overall satisfaction with the long-term follow-up was rated as “very good” by 13 patients, “good” by 10 patients, and “sufficient” by 3 adolescent pectus patients.

**Table 7:** Satisfaction with the eHealth technology for postoperative monitoring of patients at home.

Patient satisfaction of device or app^a^		Number of patients, n (%)
**Smartphone**		
	Insufficient	5 (19)
	Sufficient	6 (23)
	Good	8 (31)
	Excellent	7 (27)
**Oxygen saturation monitor**		
	Insufficient	0 (0)
	Sufficient	2 (8)
	Good	5 (19)
	Excellent	19 (73)
**Blood pressure monitor**		
	Insufficient	6 (23)
	Sufficient	6 (23)
	Good	10 (39)
	Excellent	4 (15)
**Activity tracker**		
	Insufficient	5 (19)
	Sufficient	3 (12)
	Good	6 (23)
	Excellent	12 (46)
**Sleep monitor**		
	Insufficient	3 (11)
	Sufficient	1 (4)
	Good	10 (38)
	Excellent	12 (46)
**App (daily measurements)**		
	Insufficient	1 (4)
	Sufficient	5 (19)
	Good	12 (46)
	Excellent	8 (31)
**Web-based platform (questionnaires)^b^**		
	Insufficient	0 (0)
	Sufficient	2 (8)
	Good	8 (31)
	Excellent	14 (54)
**Main reason for nonadherence**		
	Time-consuming	1 (4)
	Remembering	19 (73)
	Empty battery	2 (8)
	Device failure	4 (15)

^a^Patient satisfaction given by 26 enhanced recovery pathway patients at the final interview, 10 weeks postoperatively.

^b^Two patients did not complete this questionnaire.

## Discussion

### Principal Findings

This first implementation study evaluated different outcome variables of the implemented ERP postoperatively in early recovery and assessed the occurrence of PPSP 3 months postoperatively in pediatric patients undergoing MIRP using eHealth technology. We demonstrated the possibilities of eHealth screening and monitoring tools in a perioperative enhanced recovery program. Using Appi@Home, patients can be monitored during the entire (prolonged) rehabilitation period.

### Acute Pain and Short-Term-Related Variables

Although surgical correction of pectus deformities has been considered a minimally invasive procedure, MIRP is still accompanied by severe postoperative pain [32]. Bogert et al [33] identified pain scores of 4.1, 4.0, and 3.5 in pectus patients in the first 3 PODs, even with the PCEA treatment. Several studies have shown that postoperative pain is often difficult to manage [8,9], and higher postoperative pain scores are associated with persistent or chronic pain [3,6]. Kristensen et al [6] collected adult reports of patients after thoracotomy, and 16% of them recalled pain >3 months postoperatively. Despite pain scores for which additional treatment is some sometimes required, some physicians succeeded in early hospital discharge after 4.9 (range 3-8) days [34] or 3.1 (range 2-6) days [35]. The use of ERPs has gained major attention in recent years. However, many clinicians struggle to appropriately describe and dose postoperative analgesics while tackling the real needs of patients in acute pain [33]. Litz et al [36] recently described the potential benefit of an in-hospital ERP in patients undergoing thoracic wall deformity repair. Optimal treatment using a pre-emptive multimodal management protocol covering biopsychosocial needs improved patient-related outcome measures, whereas undertreatment of acute pain increased the risk of pain chronification [3]. Possibly, more important than the ongoing debate on the optimal peroperative and immediate postoperative treatment in the ERP (eg, epidural vs intravenous analgesia) [37], novel research suggests a more structured holistic care pathway of routine elective major surgery, understanding the relation between medication initiation, dosage, and duration, focusing on early appropriate treatment of yellow and red flags[38,39]. This requires multidisciplinary follow-up of patients, maximizing patient and parent satisfaction. Our data showed that the implementation of the ERP positively affected early rehabilitation with low pain scores, even with thorough epidural analgesia administration. Pain scores were even lower when compared with data from Litz et al who also used gabapentin but preferred early systemic opioid administration instead of epidural analgesics [36]; the scores were 5.2 (SD 1.7), 3.8 (SD 2.1), and 3.8 (SD 2.2), on POD 0, 1, and 2, respectively. Furthermore, clinicians are urged to remove chest tubes and Foley and epidural catheters as soon as possible, so that the risk of potential urinary or epidural infections and delayed rehabilitation can be reduced.

In this study, patients and their family members were instructed and educated very early in the perioperative trajectory, thereby reducing anxiety and identifying additional risk factors for increased or prolonged postsurgical pain as suggested by Williams et al using a management pathway including biopsychosocial formulation [7]. The establishment of a constructive relationship between caregiver, patient, and family, as recommended by Liossi et al [39], also provided a platform to provide perioperative context and explain interventions and expectation as indicated by patients and parents on the final interview. Furthermore, the implementation of such a holistic surgical care pathway was positively assessed by the adolescents and their parents during hospital admission as well as after discharge.

### Persistent Pain and Long-Term Rehabilitation

Our study differs from other studies in terms of the biopsychosocial evaluation and the extended daily follow-up even after hospital discharge. To date, little data concerning subacute, persistent, or chronic postoperative pain in children have been collected, despite growing knowledge regarding risk factors [7]. Our project included the recording of objective parameters, such as vital signs, and subjective variables concerning pain, daily activities, and sleep quality after hospital discharge. Hence, medical intervention could be planned early if necessary. Despite the low pain scores in our study population 3 months postoperatively, 33% (9/27) adolescents reported continued daily intake of analgesics, repeated visits to general practitioners or specialized health care services, and even school absenteeism because of thoracic neuropathic pain symptoms. The dependency of children on their parents and school absenteeism during young vulnerable life increases the importance of these numbers. A possible explanation may be that the increased body length growth or surgical correction of an asymmetrical deformity may lead to consequent increased (unilateral) pressure after fixation with potential intercostal nerve damage as suggested by Wildgaard et al [40]. However, more research with long-term evaluation is necessary to decipher causal variables.

### Implementation of eHealth and Mobile Health Care

Digital apps are on the rise in health care. The need for such apps is apparent due to the increasing tendencies toward early postoperative recovery with reduced hospital stay lengths [36,37]. Through apps, mobile technology [41], and wearables, the health of patients can be monitored more accurately and faster [42]. Consistent with our data, efficient care using this technology was positively evaluated by various patient-related outcome measurements such as pain, daily activities, and overall satisfaction [43]. In fact, mobile health can be a facilitator of evolution toward a value-based approach to care. In this first implementation trial, patients reported the monitoring tools as feasible devices, and they indicated that a rather low effort was required for Web-based questionnaire completion. However, in addition to the need to optimize the performance of the individual wearables, research should be devoted to increasing patient adherence. The use of gamification techniques and other approaches could accelerate implementation [44]. The use of such game design elements can increase the motivation of people to adhere to telemonitoring actions and Web-based questionnaires as part of their individual follow-up and therapy.

Little is known about the possibilities of eHealth in this specific patient group of pectus adolescents; however, many of them could benefit from improved perioperative care. This ERP implementation project combines various suggestions reported in other target groups such as psychological screening, structured care, and PROM. Nevertheless, more detailed research through well-designed study protocols is necessary toward postoperative (long-term) application of eHealth modalities in adolescents after a major surgery.

### Limitations

We recognize that our implementation study has some limitations. First, we compared ERP-treated patients with retrospective data in our hospital before such protocols were used for MIRP patients. Therefore, data between 2010 and 2014 were used. It should be mentioned that the Abramson technique has only been introduced in recent years. Moreover, although recognized as the most important risk factor for pain, those historical controls have only been matched for age and pathology. Furthermore, additional research is needed to further clarify the differences in multiple patient-related outcome measurements among patients treated using the ERP protocol in the 2 MIRP categories, PE and PC. Second, the adherence to the different telemonitoring devices should be further increased. The daily use of the devices is mainly diminished due to “forgot to use it”; this could be a possible explanation for the high reported activity tracker category “lying down.” Third, the design of this study focused on adolescent pectus patients without a history of opioid use or psychiatric disease. Ideally, patients diagnosed with autistic spectrum disorders or other mental illnesses should be included in an ERP, as they could benefit the most from standardized care. Our findings must, therefore, be evaluated in larger comparative descriptive studies and randomized controlled trials.

### Conclusion

Our study results offer a potential approach for optimizing holistic patient care, consequently, improving patient-reported outcome measures. Early risk factor identification and structured individual medical (long-term) follow-up after discharge may further enhance rehabilitation. Health care providers should extend their knowledge of and embrace available eHealth technologies for biopsychosocial care.

Our platform provides a framework for optimizing patient- and procedure-specific psychological Web-based screening questionnaires, individual patient monitoring, and treatment (re)assessment. Furthermore, it may contribute to scientific research by offering reliable long-term data.

The implementation of holistic surgical care pathways using a multidisciplinary eHealth-based approach is a combination that merits further investigation in various surgical patient groups.

## Appendix

Multimedia Appendix 1 Appi@Home digital platform.

Multimedia Appendix 2 Preoperative Psychological Screening Questionnaires.

Multimedia Appendix 3 Multidisciplinary Enhanced Recovery Pathway – Medication Components. PCEA, patient-controlled epidural anesthesia; TCA, target controlled anesthesia; PACU, post-anesthesia care unit; POD, postoperative day; IV, intravenous; PO, per os; PIB, programmed intermittent bolus regimen.

Multimedia Appendix 4 Standard medication reduction scheme, recommended after hospital discharge. NSAID: nonsteroidal anti-inflammatory drug. All drugs are administrated taking into account the weight of the patient.

Multimedia Appendix 5 Postoperative Psychological Questionnaires.

Multimedia Appendix 6 Vital signs during patient follow-up at home. Note that patients did not use the devices when admitted to the hospital during the early postoperative period.

Multimedia Appendix 7 Subjective outcome variables per patient during postoperative rehabilitation at home (after hospital discharge). Note that patients did not use the individual diary when admitted to the hospital during the early postoperative period. NRS: Numeric Rating Scale.

## Abbreviations

APPI: Antwerp Personalized Pain Initiative
CPQ: Coping Pain Questionnaire
eHealth: electronic health
ERP: enhanced recovery pathway
HADS: Hospital Anxiety and Depression Scale
IRB: institutional review board
LOS: length of hospital stay
MIRP: minimally invasive repair of pectus
MPI: Multidimensional Pain Inventory
NRS: Numeric Rating Scale
PC: pectus carinatum
PCEA: patient-controlled epidural analgesia
PE: pectus excavatum
POD: postoperative day
PPSP: persistent postsurgical pain
RSES: Rosenberg Self-Esteem Scale
STAI: State-Trait Anxiety Inventory
