# Supplementary material for: Implementation of an Enhanced Recovery Pathway for Minimally Invasive Pectus Surgery: A Population-Based Cohort Study Evaluating Short- and Long-Term Outcomes Using eHealth Technology
Source: JMIR Perioper Med. 2018 Oct 12;1(2):e10996. doi: 10.2196/10996 (PMC7709887; doi:10.2196/10996)
Supplement: Multimedia Appendix 1 [file periop_v1i2e10996_app1.pdf]

Multimedia Appendix 1

**Appi@Home digital platform**

Appi@Home is an award-winning eHealth telemonitoring system, allowing physicians to keep tabs on patients even after they return home. Patients can easily measure their vital parameters through our user-friendly smartphone app, as well as completing diaries or filling questionnaires. The resulting data are then made available to physicians in the Web platform, where it can be used to evaluate the patient's therapy. The app also proactively tracks down possible issues with patients and alert their physicians if necessary. Appi@Home supports our approach to subacute and chronic pain by offering a platform for continuous follow-up. Patients are provided with a toolbox and an app that continuously collects objective outcome data. Earlier (unpublished) results clearly indicate an increase in patient responsibility and self-management, with a significantly shorter treatment period and earlier functional reintegration as a result.

How it works in reality

1. Patients are asked to start using the app after the preoperative consultation when informed consent is obtained
2. Patients receive an Appi@Home box with wireless medical measuring devices.
3. The patient downloads our app "Appi@Home" for Android or iOS, which guides the patient through the set-up procedure, necessary to connect the wireless Bluetooth-connected devices with their smartphone. As an alternative, questionnaires and diaries can also be accessed by using the Web-based platform https://appi.uza.be.
4. Measurements are securely transmitted to the hospital. Data only get stored on-premises.
5. Physicians can sift through the data, and receive proactive alerts if predefined problems arise. Therefore, limits for measurement results are predefined when a caregiver is activating the platform of a patient.
6. Patients receive messages asking them to complete validated questionnaires on fixed time points, throughout the perioperative period.
