# Supplementary material for: Implementation of an Enhanced Recovery Pathway for Minimally Invasive Pectus Surgery: A Population-Based Cohort Study Evaluating Short- and Long-Term Outcomes Using eHealth Technology
Source: JMIR Perioper Med. 2018 Oct 12;1(2):e10996. doi: 10.2196/10996 (PMC7709887; doi:10.2196/10996)
Supplement: Multimedia Appendix 2 [file periop_v1i2e10996_app2.pdf]

Multimedia Appendix 2

Preoperative psychological screening questionnaires

The Hospital Anxiety and Depression Scale (HADS)

The HADS was developed for detecting states of depression and anxiety in a hospital setting [40,41]. It assesses core components of anxiety and depression without involving the physical complaints. The questionnaire has 2 subscales, anxiety and fear, and both subscales consist of 7 items. High scores indicate more emotional complaints. A validated Dutch version by Spinhoven et al was used [15].

The State-Trait Anxiety Inventory (STAI) Form Y

The STAI was used to assess state and trait anxiety [22]. In this questionnaire, state anxiety is defined as fear, nervousness, and discomfort temporarily induced by situations perceived as dangerous or threatening in which the autonomic nervous system is activated. Trait anxiety refers to rather stable individual differences in the predisposition to experience fear, stress, and discomfort. People with high trait anxiety characteristics will experience certain situations, such as surgery, as more threatening or dangerous compared with people with low trait anxiety. Van Der Ploeg developed a Dutch translation [16].

The Rosenberg Self-Esteem Scale

The Rosenberg Self-Esteem Scale (RSES) is a screening instrument for negative body image perception [42]. Self-esteem is an important measure for screening problems of social adaptation and predicting mental health problems. Furthermore, screening for body image disturbances can already be a necessary intervention in patients with a thoracic wall deformity. The RSES is a self-report measure of self-esteem containing 10 items constructed for investigating a person's feelings about themselves in terms of self-confidence and intrinsic value. The Dutch version by Franck et al was used [17].
