# Supplementary material for: Implementation of an Enhanced Recovery Pathway for Minimally Invasive Pectus Surgery: A Population-Based Cohort Study Evaluating Short- and Long-Term Outcomes Using eHealth Technology
Source: JMIR Perioper Med. 2018 Oct 12;1(2):e10996. doi: 10.2196/10996 (PMC7709887; doi:10.2196/10996)
Supplement: Multimedia Appendix 3 [file periop_v1i2e10996_app3.pdf]

Multimedia Appendix 3

The multidisciplinary enhanced recovery pathway—medication components. PCEA, patient-controlled epidural anesthesia; TCA, target controlled anesthesia; PACU, postanesthesia care unit; POD, postoperative day; IV, intravenous; PO, per os; PIB, programmed intermittent bolus regimen.

Preadmission

- Gabapentin, starting 7 days prior to surgery, < 50 kg, daily 300 mg; > 50 kg, daily 900 mg
- Day before surgery, admission to the floor, where all healthcare providers are familiar with the postoperative protocol

Day of surgery

- Multimodal analgesia
  - PCEA, optimal placement T8-T10. Ropivacaine bolus 3.75 $mgml^{-1}$, 1 ml per 10 cm above 1 m patient length
  - IV Acetaminophen 20 $mgkg^{-1}$, 15 $mgkg^{-1}$ repeated every 6 hours
  - IV Ketorolac 0.5 $mgkg^{-1}$
- Multimodal antiemetics
  - IV Dexamethasone 5 mg
  - IV Dehydrobenzperidol 0.625 mg
  - IV Ondansetron 4 mg
  - TCA Propofol
- Chest tube and Foley catheter placement
- When awake: incentive spirometry as frequently as possible
- PACU admission overnight: continuous pulse oximetry and supplemental oxygen if necessary to maintain saturation
- Stimulation early intake as tolerated

POD 1 - 2

- PCEA mixture ropivacaine 0.2 % + fentanyl 10 μgml^{-1}, 6 ml/h continuously, 4 ml/h bolus if necessary every 30 minutes (6/4/30) – coadministered with ondansetron 4 mg every 8 hours
- IV Acetaminophen 15 $mgkg^{-1}$ repeated every 6 hours
- IV Ketorolac 0.5 $mgkg^{-1}$ every 8 hours – coadministered with ranitidine 2–4 mg/kg/day
- Continue gabapentin administration according to preoperative scheme
- Laxative association
- Early mobilization (supine position POD 1, sitting and standing position POD 2) and incentive spirometry
- Remove chest tube POD 1 after thoracic X-ray, remove Foley catheter if PCEA settings have been reduced on POD 2

POD 3

- PCEA (ropivacaine 1.6 % + fentanyl 10 μgml^{-1}) setting reduction 4/3/10 - coadministered with ondansetron 4 mg every 8 hours
- Convert to PO acetaminophen and NSAID
- Continue gabapentin and laxative administration
- Intensify mobilization (standing position and walking) and incentive spirometry
- If not already done, remove Foley catheter

POD 4

- PCEA (ropivacaine 1.6 % + fentanyl 10 μgml^{-1}) setting reduction PIB 5/120/3/10
- Escape buprenorphine 0.2 mg if necessary for breakthrough pain during PCEA weaning
- Continue PO medication
- Increasing mobilization and rehabilitation

POD 5

- PCEA (ropivacaine 1.6 % + fentanyl 10 μgml^{-1}) setting reduction 0/3/10
- Escape buprenorphine 0.2 mg if necessary for breakthrough pain during PCEA weaning
- Start tilidine/naloxone slow release 50 mg every 12 hours in preparation for PCEA discontinuation
- Continue PO medication
- Evaluates patient for completion of activities of daily living

POD 6

- If not already done, discontinue PCEA and ondansetron administration
- Stop NSAID and coadministration of ranitidine
- Discharge patient when
  - pain is well controlled on PO medication,
  - given education on medication reduction scheme and long-term eHealth follow-up,
  - emphasize physical activity exercises and precautions preventing implanted material dislocation
