# Supplementary material for: Implementation of an Enhanced Recovery Pathway for Minimally Invasive Pectus Surgery: A Population-Based Cohort Study Evaluating Short- and Long-Term Outcomes Using eHealth Technology
Source: JMIR Perioper Med. 2018 Oct 12;1(2):e10996. doi: 10.2196/10996 (PMC7709887; doi:10.2196/10996)
Supplement: Multimedia Appendix 4 [file periop_v1i2e10996_app4.pdf]

Multimedia Appendix 2

Standard medication reduction scheme, recommended after hospital discharge

NSAID: non-steroidal anti-inflammatory drug. All drugs are administrated taking into account the weight of the patient.

Hospital discharge

- Stop NSAID and coadministered ranitidine

After 7 days

- Reduce tilidine/naloxone slow release 50 mg two times a day to one a day regimen, earlier reduction is recommended if excessively sleepy.
- Continue acetaminophen

After 12 days

- Reduce gabapentin from three times a day, to twice a day
- Continue acetaminophen

After 13 days

- Reduce gabapentin from two times a day, to once a day
- Continue acetaminophen

After 14 days

- Stop tilidine/naloxone
- Stop gabapentin
- Acetaminophen can be administrated if necessary
