# Supplementary material for: Implementation of an Enhanced Recovery Pathway for Minimally Invasive Pectus Surgery: A Population-Based Cohort Study Evaluating Short- and Long-Term Outcomes Using eHealth Technology
Source: JMIR Perioper Med. 2018 Oct 12;1(2):e10996. doi: 10.2196/10996 (PMC7709887; doi:10.2196/10996)
Supplement: Multimedia Appendix 5 [file periop_v1i2e10996_app5.pdf]

Multimedia Appendix 5

Postoperative psychological questionnaires

Multidimensional Pain Inventory (MPI)

The Dutch version of the (West Haven Yale) Multidimensional Pain Inventory (MPI) was used to assess different pain-relevant aspects [19,24]. The first part used in this project assesses the psychosocial aspects of pain and consists of 5 subscales—pain severity, interference, life control, affective distress, and social support.

Coping with Pain Questionnaire (CPQ)

The second postoperative questionnaire is the Dutch version of the Coping Strategies Questionnaire (CSQ), the Coping with Pain Questionnaire (CPQ), which was developed by Spinhoven et al [25]. The CPQ contains 44 items in 8 subscales—diverting attention, reinterpreting pain sensations, using coping self-statements, ignoring pain sensations, praying/hoping, catastrophizing, increased behavioral activities, and perceived control over pain. The respondent answers questions on a visual analog scale (CPQ) instead of a 7-point Likert-type scale (CSQ). CPQ active and passive coping indices were calculated according to the method described by Soares and Grossi [43] and Nicholas et al [44]. The scores of 5 subscales that reflect active coping were calculated to determine an active coping index. Two scales that refer to passive coping were used to create a passive coping index.
