# Supplementary figures and images for: Implementation of an Enhanced Recovery Pathway for Minimally Invasive Pectus Surgery: A Population-Based Cohort Study Evaluating Short- and Long-Term Outcomes Using eHealth Technology
Source: JMIR Perioper Med. 2018 Oct 12;1(2):e10996. doi: 10.2196/10996 (PMC7709887; doi:10.2196/10996)

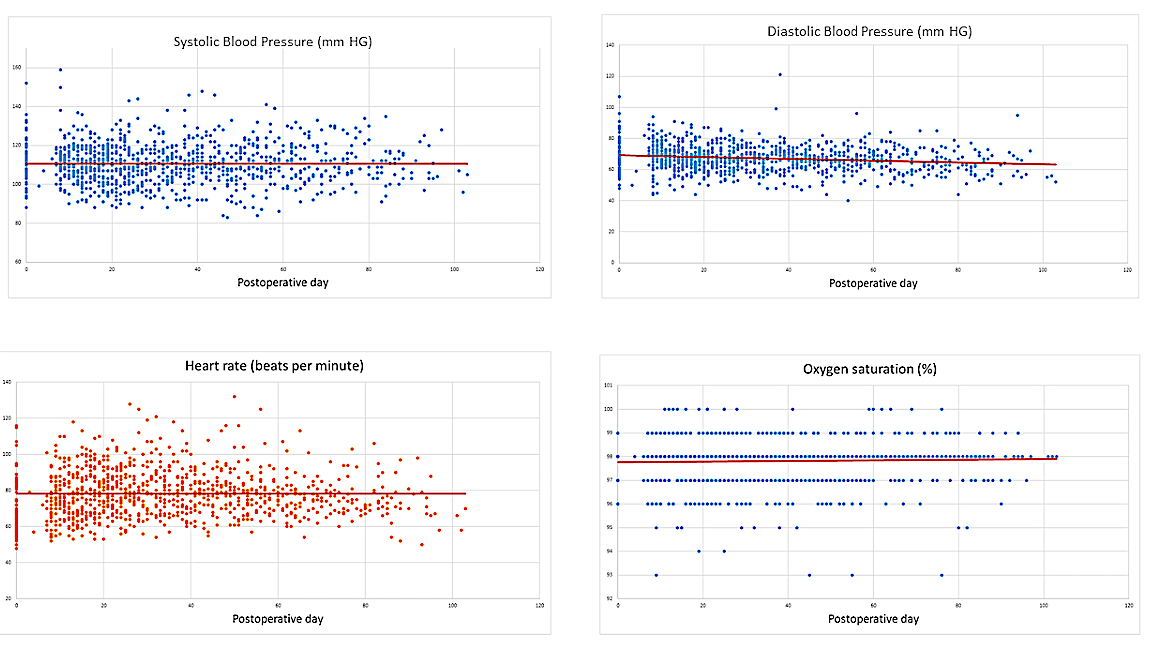

Supplement: Multimedia Appendix 6 [file periop_v1i2e10996_app6.png]

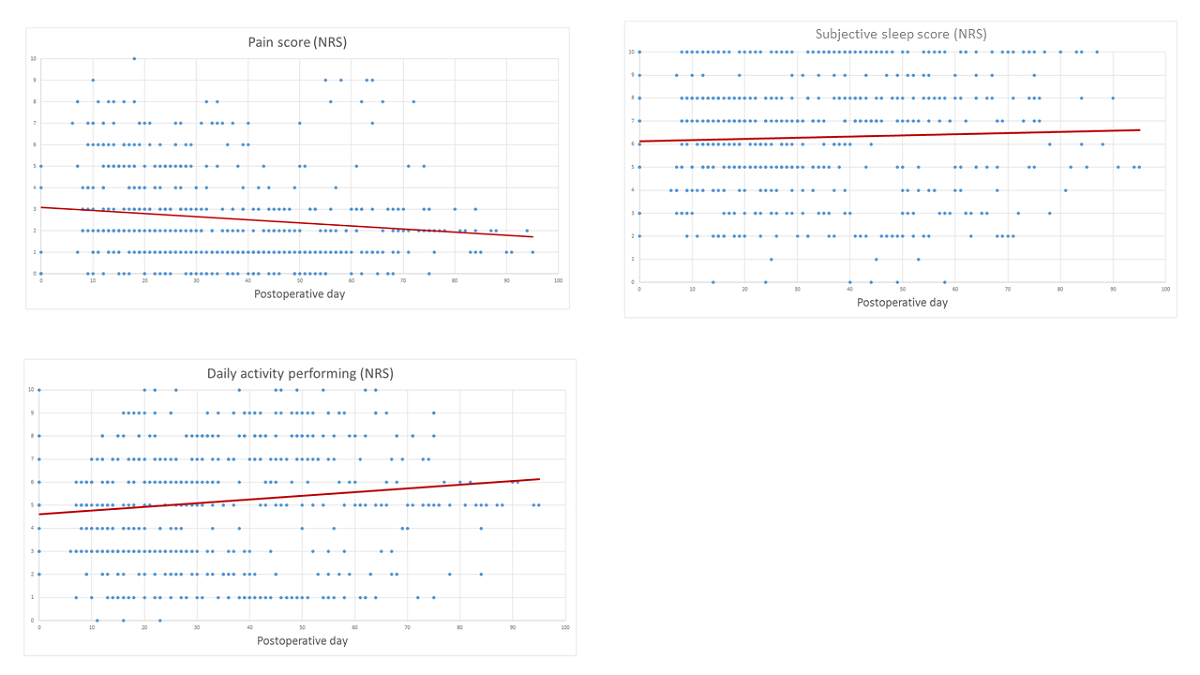

Supplement: Multimedia Appendix 7 [file periop_v1i2e10996_app7.png]
